# Perforation Peritonitis and the Developing World

**DOI:** 10.1155/2014/105492

**Published:** 2014-04-02

**Authors:** Rajandeep Singh Bali, Sushant Verma, P. N. Agarwal, Rajdeep Singh, Nikhil Talwar

**Affiliations:** ^1^Department of General Surgery, Lok Nayak Hospital, New Delhi 110002, India; ^2^Department of General Surgery, Maulana Azad Medical College and Lok Nayak Hospital, New Delhi 110002, India

## Abstract

*Background*. Perforation peritonitis is the one of the commonest emergency encountered by surgeons. The aim of this paper is to provide an overview of the spectrum of perforation peritonitis managed in a single unit of a tertiary care hospital in Delhi. *Methods*. A retrospective study was carried out between May 2010 and June 2013 in a single unit of the department of Surgery, Lok Nayak Hospital, Delhi. It included 400 patients of perforation peritonitis (diffuse or localized) who were studied retrospectively in terms of cause, site of perforation, surgical treatment, complications, and mortality. Only those patients who underwent exploratory laparotomy for management of perforation peritonitis were included. *Results*. The commonest cause of perforation peritonitis included 179 cases of peptic ulcer disease (150 duodenal ulcers and 29 gastric ulcers) followed by appendicitis (74 cases), typhoid fever (48 cases), tuberculosis (40 cases), and trauma (31). The overall mortality was 7%. *Conclusions*. Perforation peritonitis in India has a different spectrum as compared to the western countries. Peptic ulcer perforation, perforating appendicitis, typhoid, and tubercular perforations are the major causes of gastrointestinal perforations. Early surgical intervention under the cover of broad spectrum antibiotics preceded by adequate aggressive resuscitation and correction of electrolyte imbalances is imperative for good outcomes minimizing morbidity and mortality.

## 1. Introduction

Gastrointestinal perforations constitute one of the commonest surgical emergency encountered by surgeons [1, 2]. Management of these patients continues to be highly demanding despite the advances made in diagnosis and surgical therapy. The etiological spectrum of perforation peritonitis in India differs significantly from its western counter parts [3–5]. Our study was carried out to highlight the spectrum of perforation peritonitis (diffuse) in a single unit at Lok Nayak Hospital, a tertiary care hospital in Delhi.

## 2. Patients and Methods

The retrospective study was conducted at the Department of Surgery, Maulana Azad Medical College and associated Lok Nayak Hospital, Delhi, from May 2010 to June 2013. The study population included 400 patients of perforation peritonitis (diffuse or localized) presenting to the surgical emergency of Lok Nayak Hospital, Delhi, who underwent exploratory laparotomy.

Cases were studied with respect to clinical features at the time of presentation, comorbid conditions, radiological investigations, operative findings, and postoperative course. After establishing the clinical diagnosis of perforation peritonitis the patients were prepared for exploratory laparotomy. On performing exploratory laparotomy, the operative findings were noted and the source of peritonitis was found and managed accordingly. All patients were then treated in the postoperative ward initially under the cover of parenteral broad spectrum antibiotics and fluids; orals were started on the appearance of bowel sounds.

## 3. Results

A total of 400 patients were studied. Mean age was 37.8 years (range from 13 to 88 years). Majority of patients were males (68.5%). Male: female ratio was 2.1 : 1, respectively. 98% patients presented with the history of abdominal pain, 62.5% with altered bowel habit, 41.5% with nausea and vomiting, and 28% with abdominal distention. 15% patients had positive history of NSAID intake for more than 6 months (Table 1).

In our study, 15% cases had associated comorbid conditions. The commonly associated comorbidity was chronic obstructive pulmonary disease followed by renal disease, diabetes, and hypertension.

79% patients had pneumoperitoneum on chest X-ray in erect posture. Multiple air fluid levels on abdominal X-ray in erect position were present in 28% patients. Electrolyte imbalances included hyponatremia in 21%, hypokalemia in 19% and elevated serum creatinine in 18% patients. Most of the patients were operated within 24 hours of presentation under the cover of broad spectrum antibiotics after adequate resuscitation and correction of electrolyte imbalances.

The commonest cause of perforation peritonitis in our study was gastroduodenal perforation due to acid peptic disease (45%) followed by appendicitis (18.5%), typhoid fever (12%), tuberculosis (10%), and trauma (9%), (Table 2).

Patients of peptic ulcer perforation usually had a short history of pain starting in epigastrium followed by generalized tenderness. 34% of these patients had history of NSAID intake for >6 months. 175 such were managed by an omental pedicle repair, in the other 4 cases a feeding jejunostomy was also done due to the large size of the perforation.

Patients with appendicular perforation presented with right iliac fossa pain along with localized peritonitis. 8% of these patients were managed by a limited resection with Ileo-ascending anastomosis due to associated unhealthy caecum.

Patients of typhoid perforation had an initial history of high grade fever prior to abdominal complaints. 83% were located in the ileum and 40% were multiple.

Of the 40 patients of tubercular perforation, 60% had previous history of tuberculosis and 50% of these patients took antitubercular therapy for <6 months.

In cases of traumatic perforation, the most common site was jejunum (49%) followed by ileum (42%).

The most commonly performed procedure was omental pedicle closure of peptic ulcer perforation (43.75%), followed by exteriorization of the gut in the form of ileostomy or colostomy (22.5%). Appendectomy was the third most common procedure (17%), (Table 2).

189 out of 400 cases developed postoperative complications (Table 3).

The most common complication was wound infection followed by dyselectrolytaemia, abdominal collection, and respiratory complications. The morbidity rate was higher in the patients with intestinal perforation (58%) than those with gastroduodenal perforation (32%).

The overall mortality rate was 7%. Factors involved in death included septicemia due to fecal peritonitis, respiratory complications, pulmonary embolism, and late presentation.

## 4. Discussion

One of the most common surgical emergencies is perforation peritonitis [6]. It is commonly seen in a younger age group in the tropical countries (mean age in our study was 37.8 years) as compared to the studies in the West [7–9].

More commonly the perforations involve the proximal part of the gastrointestinal tract; [10–13] this being in contrast to studies from the western countries, where perforations are common in the distal part [14–16].

Etiological factors also show a wide geographical variation. According to a study from India, infections formed the most common cause of perforation peritonitis [17], around 50% cases in this study were due to typhoid. In our study 22% of the cases were due to typhoid and tuberculosis. In contrast to this, Noon et al. [18] from Texas in their study reported only 2.7% cases due to infections. Also studies from the west have shown that around 15–20% cases are due to malignancy [19, 20], this being in stark contrast to our study where malignancy was ascertained to be the cause of perforation peritonitis in only 3% of the cases. This shows that malignancy is not a common cause of perforation peritonitis in our setup as compared to our western counterparts.

The overall mortality due to perforation peritonitis ranges between 6 and 27% [21]. The mortality rate in our study was 7%. One of the most important factors responsible for mortality is septicemia. Adequate preoperative resuscitation (with fluids, etc.), correction of electrolyte imbalances followed by an early surgical intervention, to remove the source of infection and stop further contamination, is imperative for good outcomes minimizing morbidity and mortality.

## 5. Conclusion

Perforation peritonitis in India has a different spectrum as compared to the western countries. Peptic ulcer perforation, perforating appendicitis, typhoid, and tubercular perforations are the major causes of gastrointestinal perforations. Early surgical intervention under the cover of broad spectrum antibiotics preceded by adequate aggressive resuscitation and correction of electrolyte imbalances is imperative for good outcomes minimizing morbidity and mortality.

## Figures and Tables

**Table 1:** Preoperative data [sex, comorbid conditions, and signs and symptoms].

Parameter		*N*
Sex	Male	274 [68.5%]
	Female	126 [31.5%]

Comorbid conditions	Respiratory disease	60
	Diabetes mellitus	41
	Renal disease	36
	Hypertension	16

Symptoms and signs	Abdominal pain	392 [98%]
	Altered bowel habit	250 [62.5%]
	Nausea and vomiting	166 [41.5%]
	Abdominal distention	112 [28%]
	Positive H/O NSAID (>6 months)	61 [15.25%]
	Tachycardia (pulse > 100/minute)	122 [30.5%]

**Table 2:** Etiology and site of perforations.

Parameter		*N*
Causes of perforation	Acid peptic disease	179 [44.75%]
	Appendicitis	74 [18.5%]
	Typhoid	48 [12%]
	Tuberculosis	40 [10%]
	Trauma	31 [7.75%]
	Malignancy	13 [3.25%]
	Bowel strangulation	8 [2%]
	Band obstruction perforation	4 [1%]
	Amoebic caecal perforation	3 [0.75%]

Site of perforation	Duodenum	150 [37.5%]
	Ileum	90 [22.5%]
	Appendix	74 [18/5%]
	Jejunum	38 [9.5%]
	Stomach	29 [7.25%]
	Sigmoid colon	8 [2%]
	Caecum	5 [1.25%]
	Transverse colon	3 [0.75%]
	Descending colon	3 [0.75%]

Surgical procedure	Omental patch	175 [43.75%]
	(With feeding jejunostomy)	(4)
	Stoma	90 [22.5%]
	Appendectomy	68 [17%]
	Primary repair	27 [6.75%]
	Resection-anastomosis	25 [6.25%]
	Limited resection with Ileo-ascending anastomosis	6 [1.5%]
	Right hemicolectomy	5 [1.25%]

**Table 3:** Postoperative complications.

Complication	*N*
Wound infection	125 [31.25%]
Dyselectrolytaemia	87 [21.75%]
Abdominal collection	80 [20%]
Respiratory complication	67 [16.75%]
Burst abdomen	55 [13.75%]
Anastomotic leak	6 [1.5%]
Mortality	28 [7%]
